# Simulation-based assessment of the soil organic carbon sequestration in grasslands in relation to management and climate change scenarios

**DOI:** 10.1016/j.heliyon.2023.e17287

**Published:** 2023-06-17

**Authors:** Matthias Filipiak, Doreen Gabriel, Katrin Kuka

**Affiliations:** a Julius Kühn Institute (JKI) – Federal Research Centre for Cultivated Plants, Institute for Crop and Soil Science, Bundesallee 58, 38116 Braunschweig, Germany; b Thuenen Institute of Agricultural Technology, Bundesallee 47, 38116 Braunschweig, Germany

**Keywords:** Soil organic carbon, Simulations, Management regimes, Future climate scenarios, Grassland

## Abstract

Soil organic carbon (SOC) is crucial for the quality and productivity of terrestrial ecosystems and its sequestration plays an important role in mitigating climate change. Understanding the effects of agricultural management under future climate on the SOC balance helps decision making in environmental policies. Thereby, grasslands will play a key role, since future climate change may prolong the vegetation period.

We used 24 representative grassland sites in Germany to assess the SOC balance obtained from the CANDY model in relation to ten management regimes, 18 future climate change scenarios and different soil types. Simulations were conducted over a period of 110 years.

For most of the selected grassland sites an increase in both air temperature and precipitation was observed in the future climate. The effect of management on the SOC balance largely exceeded the effect of soil type and climate. An increasing management intensity (i.e. three to five cuts) generally increased the SOC balance, while extensive management (i.e. two or fewer cuts) lead to SOC losses. The seasonal variation of precipitation was the most important climate metric, with increased SOC sequestration rates being observed with increasing growing season precipitation. Clay soils had the potential for both highest gains and highest losses depending on management and precipitation. Given an overall lower SOC storage potential in sands and loams, the SOC balance in those soil types varied the least in response to climate change.

We conclude that fostering SOC sequestration is possible in grassland soils by increasing management intensity, which involves increased fertilizer input and field traffic. This however may stand in conflict with other policy aims, such as preserving biodiversity. Multicriterial assessments are required to estimate the nett greenhouse gas balance and other aspects associated with these management practices at a farm scale.

## Highlights

• SOC-Simulation based on management and climate scenarios on representative grasslands.
• Effect of management on SOC sequestration was stronger than soil type and climate.
• Highest SOC gains occurred under four to five cuts; two or fewer resulted in losses.
• SOC gains under four cuts and losses under two cuts were highest on clays.
• Principal climate metric promoting sequestration was growing season precipitation.

## 1 Introduction

In a resolution from March 24, 2021, Germany's Federal Constitutional Court established a new goal of reducing agricultural greenhouse gas (GHG) emissions by 34% by the year 2030, in comparison to the reference year 1990, and achieving climate neutrality by 2045 [1]. Sequestration of soil organic carbon (SOC), i.e. the removal of carbon dioxide (CO_2_) from the atmosphere and increasing the C pool size in soil and vegetation, is a central strategy for the mitigation of GHG emissions [2]. Recent studies show the importance of identifying potential synergies between land-based adaptation and mitigation strategies, linking issues of SOC sequestration with emissions of GHGs and long-term sustainability of production systems within coherent climate policy frameworks [[2], [3], [4], [5], [6]]. The soil C cycle is furthermore central to the functioning of terrestrial ecosystems [4,[7], [8], [9], [10], [11]] and high SOC contents are also beneficial to soil functions relevant for agriculture, such as the regulation of nutrient-, water- and temperature flows and the soil structure [[12], [13], [14], [15], [16]]. Due to their year-round plant cover, high biodiversity and usually undisturbed soil, grassland areas are important ecosystems for SOC sequestration [10,[17], [18], [19], [20], [21], [22], [23]].

For steady state soil systems, the equilibrium between C input and release results in a characteristic SOC content depending on local environmental conditions [24], e.g. management, climate and soil properties [15,25,26]. There is evidence suggesting a prevalence of management-related effects (i.e. cutting frequency, grazing intensity, fertilization) on SOC sequestration over that of soil type and climate [15,19,27,28]. Therefore, it is both possible and necessary to adapt management practices, e.g. by increasing the cutting frequency, grazing regimes or fertilizer input, to regional climatic conditions expected in the future decades in order to increase the sequestration potential of grassland soils [[29], [30], [31], [32]]. Issuing of CO_2_ certificates can provide the necessary incentive for farmers to implement such management practices [33]. However, the magnitude of the individual effects varies substantially across soil types and climates. It is therefore crucial to identify soil characteristics and management practices that display a high potential for long-term SOC sequestration under a broad spectrum of climatic conditions [3,31].

The analysis of long-term SOC sequestration requires prognostic approaches. Hence modelling the terrestrial C cycle has become a major research topic, which allows identifying key drivers for SOC sequestration and thereby making management recommendations based on present and expected future conditions. Process oriented ecosystems models that account for the major processes and interactions between various components of the ecosystems are the best available tools to increase our understanding and to make descriptions of impacts of climate change and its variability [3]. Models like Roth-C [34], DNDC [35], MONICA [36], DAYCENT [37], PaSim [38], ecosys [39], ECOSSE [40,41] and CANDY [42] have been developed in order to simulate long-term SOC turnover and its stabilization in the soil. Their application allows a scenario-based evaluation of management practices and their impact on terrestrial C cycles as well as the identification of preferential sites with high potential of long-term SOC storage. Several studies already conducted precise assessments of soil productivity, SOC dynamics and stocks on a grid-level up to a resolution of 1 km on regional, national and even continental scales, thereby substantially contributing to the assessment of the global sequestration potential (e.g. Refs. [3,[43], [44], [45], [46], [47]]). Nevertheless, these studies rarely investigate changes under future climate and those that do (i.e. [3,43,45,46]), consider only few climate scenarios, thereby limiting the robustness of the results to the uncertainty of future climate change, and furthermore lack multiple management variants considered per simulation unit, thereby not providing any suggestions to management alternatives. Other simulation studies dedicated to specifically investigating the interactions of climate effects across multiple sites and management practices are limited either by a narrow gradient of management regimes considered, lack of future climate scenarios, too few sites considered or lack of replication of different management regimes under varying pedo-climatic conditions (e.g. Refs. [[48], [49], [50], [51]]).

This study is motivated by the necessity to amplify the SOC sequestration in agriculture in order to meet the goals imposed by global and national policies [1,2] and provides a foundation for political decision-makers for management practices that are most suited to these goals under the predicted future climate change. The focus of this study is on the investigation of the SOC balance in grassland soils depending on soil characteristics, management intensity and climate change over a long period using the CANDY model. Validations of CANDY-based simulations of several ecosystem functions, including SOC sequestration [52,53], water quality [54] and lateral as well as vertical nutrient transport [55] were successfully conducted on various international long-term field experiments and study sites. In order to increase the national coverage and to account for uncertainties in the predicted SOC sequestration that arise from future climate scenarios [56], simulations were conducted across 24 representative grassland sites in Germany by applying 18 site-specific representative climate pathways (RCP) scenarios [57] and ten management regimes over a period of 80 years (2020–2099), including a baseline simulation of 30 years under ambient climate (1990–2019), which allows for a steady-state initialization of the SOM pools under conditions representative of each site.

The simulations aimed at testing the following hypotheses:

1. The impact of climate change on the SOC balance depends on the soil type.
2. Increasing management intensity can enhance the SOC sequestration rate.
3. The effect of management on the SOC balance prevails regardless of soil types and climate.

In the following sections, we first introduce the CANDY model, its core concepts and the model initialization process. We then describe the data used for parametrization, validation and scenario simulations and present our approach of the selection of future scenario sites, derivation of representative management regimes and future climate scenarios. Then, the initialization of the future scenario simulations is described, followed by a section outlining the statistical evaluation based on the multimodel inference approach [58]. We present the results of the model parametrization, validation and the change of the topsoil SOC contents in the future scenario period (2020 to 2099) in dependence of site properties (soil type, management, climate). We finally identify essential drivers that promote SOC sequestration as well as site conditions with the highest potential for sequestration and site conditions with the highest risk of SOC losses under future climate change.

## 2 Material and methods

**Fig. 1:**
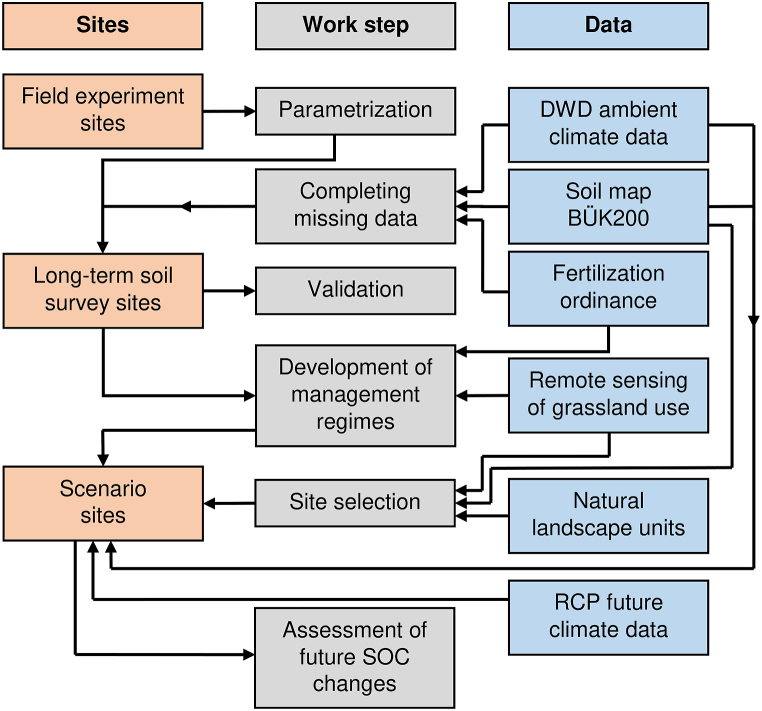
Simplified flowchart of all work involved in the assessment of the sequestration potential. The work steps are ordered sequentially from top to bottom. Arrows indicate the direction in which results or data were used for later work steps or sites. Field experiments served the parametrization, which was validated based on long-term soil survey sites. Data gaps in validation sites were filled using the BÜK200 soil map [dataset] [59], the DWD Climate Data Centre [[60], [61], [62]] and the German fertilization ordinance [63]. Management regimes were developed based on the fertilization ordinance, remote sensing data [dataset] [[64], [65], [66]] and data from the validation sites. Scenario sites were selected based on the remote sensing data, the BÜK200 and natural landscape units [67]. Input data for scenario sites was acquired from the BÜK200 [59], DWD Climate Data Centre [[60], [61], [62]] and RCP scenarios [57,[68], [69], [70], [71],^72^]. Future SOC changes were assessed based on the scenario sites.

The general work process encompassed the re-parametrization of the CANDY model with field experiment data, validation with long-term soil survey sites, derivation of management regimes, selection of scenario sites based on a variety of digital map data and subsequent simulation and evaluation of future SOC balance (Fig. 1).

### 2.1 CANDY model

CANDY [42,52] is a deterministic, one-dimensional mathematical process model that can simulate the C- and N-dynamics, soil temperature and soil water dynamics in a soil profile of the unsaturated zone of arable land and grassland up to a daily resolution. In CANDY, the concept of biological active time (BAT) is implemented to determine the site-specific turnover conditions in the soil, which reflect on the impact of temperature, soil moisture and aeration of the soil on the SOC turnover [73]. Therefore, this model is particularly useful to evaluate the effect of climate scenarios on SOC turnover. The soil profile to be simulated is divided into homogeneous horizons up to a depth of 200 cm. Simulations are conducted based on a series of fixed generalized parameters (e.g. plant development characteristics, nutrient turnover- and transport rates) as well as site-specific drivers, i.e. soil properties, meteorological data and agricultural management data [74]. For grassland simulations using CANDY the following input data are required: 1) Annual land use information (sowing date; beginning and end of grazing, number of cattle units; type, date and amount of fertilizers applied; cutting date and yield), 2) daily climate data (air temperature; precipitation; global radiation or sunshine duration) and 3) physico-chemical soil properties of each homogenous soil horizon (soil texture; bulk (BD) and particle density (PD); rock content; water content at permanent wilting point (PWP) and field capacity (FC); saturated hydraulic conductivity (Ks); specific heat capacity (Hc)).

The sowing event specifies the cultivar and land use of any given simulation unit. Due to the continuous nature of the simulated permanent grassland, the sowing event is solely required for the site initialization.

The physico-chemical soil properties for each site were assumed static, since they were derived from generalized map data. Internal pedotransferfunctions in the CANDY model (PTFs, [42,52,75]) allow the estimation of missing soil properties (e.g. PWP, FC, Ks) based on easily available properties (e.g. texture, BD). The SOM pools within CANDY are by default initialized in a steady state. The calculation of the inert C content was always done with the equation “particle surface” according to Refs. [74,76], which was identified as a reliable method to initialize the long-term-stabilized pool of the model (see also [77,78]).

The initial value of the SOC content and its division into the convertible pool and the inert pool at the start of the modelling have a decisive impact on the simulation results especially in short periods of few years [78,79]. This requires a high precision of the initial value, which does not always apply to measured field data due to systematic or methodological errors of SOC measurements. In addition, a measured SOC value is not always available when starting the simulation. Hence, to initialize the model, the CANDY Optimizer Tool in the version 20.15.2.2 [80] was used for the parametrization and validation. An optimized starting value was determined by a stepwise modification by means of several simulation runs (typically up to 50) until the root mean square error (RMSE) was minimized. This procedure allows to reduce the statistical error of simulations and bring the start of the simulation forward when necessary [81]. Combined with the steady-state initialization and partition of the C pools as outlined above, this procedure allows to begin simulations with the period of interest without the requirement to account for past management (see also [77,78]). Due to its prior focus on arable land, selected parameters within the CANDY model (i.e. the transpiration coefficient, the duration of the vegetation period and the C/N-ratio in fresh organic matter) were re-parametrized to increase its accuracy and robustness on grassland sites.

### 2.2 Data availability

#### 2.2.1 Field experiments

For the parametrization, datasets including the management history, climate and pedogenic properties from three field experiments were used: 1) the Global Change Experimental Facility in Bad Lauchstädt (GCEF, 20 plots with 4 years of SOC measurement data each, [82]), 2) the Free Air CO_2_ Enrichment experiment in Giessen (GiFACE, 3 plots with 21 years of data each, [83]) and 3) the Jena Experiment (5 plots with 7 years of data each, published in the PANGAEA network [dataset] [[84], [85], [86], [87], [88], [89]]). These data are particularly suitable for parametrization since they were measured under controlled management conditions (i.e. similar dates of operations, similar fertilizer input under all replicated plots) that remain largely constant across the entire duration of the field experiment and represent a high level of detail.

#### 2.2.2 Long-term survey sites

The validation was carried out with data from long-term survey grassland sites from Lower Saxony (14 sites with a total of 256 years of SOC measurement data), Brandenburg (3 sites, 126 years of data) and Baden-Württemberg (6 sites, 137 years of data). The sites are spread across a variety of soil types and climatic regions in Germany. These data were also used for the derivation of management regimes for the scenario simulations, alongside the datasets of long-term survey sites from Thuringia (4 sites, 94 years of data), Bavaria (23 sites, 610 years of data), Saxony (2 sites, 19 years of data) and Hesse (7 sites, 111 years of data).

#### 2.2.3 Map data

A broad variety of digital geographic map material served as basis for the selection of scenario sites, derivation of soil properties and management regimes, using Arc GIS 10.3 [90]. The map data include 1) the open-access national soil map BÜK200 [dataset] [59], 2) a digitalized climate landscape map [67] and 3) remote sensing data on grassland use, which is also available online [dataset] [[64], [65], [66]]. The [dataset] BÜK200 contains 1) site-specific information in a scale of 1:200.000 on soil system unit, soil region, the soil type including physical and chemical soil properties according to the German classification system provided by the KA5 soil mapping guideline [91] and 2) land use information. CANDY simulations based on soil maps were proven viable in previous studies [79]. The climate landscape map provides a classification of 506 natural landscape units within Germany in a scale of 1:200.000. The remote sensing data comprises raster data across Germany on 1) grassland use in the year 2016, 2) the annual number of cuts for 2017 until 2019 and 3) cutting dates for 2018 and 2019 in a spatial resolution of 30 m [dataset] [[64], [65], [66]]. These digital map data provide soil data and management information in a sufficient spatial resolution for the application of the CANDY model.

#### 2.2.4 Selection of scenario sites

For the selection of adequate and representative scenario sites for future climate simulations, several criteria were set. On a first level, the largest possible national coverage and representativeness of both climatic regions and soil types by the selected simulation sites were sought. For this purpose, the natural landscape units were grouped by soil regions. This was achieved by assigning each of the 506 polygon units of the natural landscape map to one of 12 soil regions according to BÜK200 by the largest area share the soil region takes up in the respective natural landscape.

In the next step, within each grouping of natural landscapes by soil region, the two largest natural landscape units were selected. A scenario site was determined within a selected natural landscape unit which simultaneously fulfils the following requirements: 1) it was under continuous, long-term grassland use according to the available grassland masks of remote sensing analyses from 2016 to 2019 [dataset] [[64], [65], [66]] as well as land-use information provided by the BÜK200 and 2) it is the most widespread soil type of grassland in the selected natural landscape unit (excluding organic soils) according to the BÜK200.

#### 2.2.5 Scenario climate data

The required ambient climate data (1990–2019) as model input were acquired from climate stations in the closest proximity to the selected scenario sites (see supplementary material S1 for a detailed summary of ambient climate data, including site coordinates and weather stations). For the future climate (2020–2099), site-specific data from RCP (representative climate pathways) scenarios, which were created as part of the IPCC Fifth Assessment Report [57] and served as basis for the creation of the recent SSP (shared socioeconomic pathways) scenarios [92], were used. While the recent SSP scenarios describe different socioeconomic pathways that could lead to different levels of GHG emissions as an effect of population growth, economic development, energy use, and technological change, the RCP scenarios quantify the increase in the radiative forcing in comparison to pre-industrial times by 2.6, 4.5 and 8.5 W m^−2^, which corresponds to an increase in CO_2_-equivalents to 490, 650 and 1370 ppm respectively by the year 2100, resulting in different patterns of precipitation, air temperature and global radiation. The RCP scenarios do not explicitly consider socioeconomic factors or policy decisions and avoid uncertainties associated with assumptions about socioeconomic drivers, making them useful to assess the potential physical impacts of climate change.

Each RCP scenario is realized by multiple projections resulting from simulations of different climate models and are therefore possible outcomes of future climate conditions [57]. In order to reduce the impact of climate model-specific errors and uncertainties, the selection should include as many projections (i.e. climate models) per RCP scenario as possible. From all available climate models, those models were selected that provided site-specific climate data (global radiation, precipitation, air temperature) for each day of the year in all three RCP scenarios (see supplementary material S2 for list of climate models used for future data and supplementary material S3 for a summary of future climate data). A detailed description of the RCP scenarios can be found in Refs. [[68], [69], [70], [71], [72], [93]]. The raw data can be accessed at the [dataset] [72].

#### 2.2.6 Closing data gaps

The validation data contained various gaps concerning climate, soil and management data, which were closed using a variety of open access sources as described below.

A total of 10.6% of climatic data (largely sunshine duration; see also supplementary material S4a) were completed using the online and open-access DWD Climate Data Centre [[60], [61], [62]]. Site-specific coordinates of the grassland sites served to identify the spatially closest climate stations for each site. In some cases, data from more than one climate station had to be used, if the closest climate station did not provide a complete data set.

Management data of the validation sites displayed partly large gaps (35.8%) due to the descriptive character of provided management information (see also supplementary material S4b). Missing dates for management operations, such as cutting, grazing and fertilization, were completed with available long-term means for the respective site. Missing stocking densities, fertilizer input and yield were derived from long-term means and descriptive management documentations of the original dataset while considering yield levels and nitrogen (N) requirements based on the German fertilizer ordinance [63], which is the state of the art guideline for agricultural practices.

18.9% of soil data (see also supplementary material S4c), largely for subsoil horizons, were initially missing. Texture, BD and rock contents for the validation sites were completed with the BÜK200 soil map by using site-specific coordinates. Therein, the soil profile parametrizations were provided as classes, grouped by specific ranges of the properties, according to the soil mapping guideline KA5, which is predominantly used across Germany [91]. For each class of sand-, silt-, clay- and rock content as well as BD the median between the upper and lower delimiter was used. The BÜK200 usually provides several parametrizations per location. From all available parametrizations, we chose the one that shared the same soil type according to the KA5 classification with the to-be-completed soil and, if possible, was designated as a grassland soil within the BÜK200. If no parametrization with the same soil type was available, the most similar soil type based on expert knowledge was selected instead. Missing data on the PWP, FC and Ks were acquired by using the PTFs integrated within the CANDY model. For Hc the standard value of 0.16 J cm^−3^ K^−1^ was assumed, which proved reliable in prior simulations [42,52,75]. Similarly, a PD of 2.6 g cm^−3^ was assumed for all horizons due to the predominance of quartz in soil particles [20,94], which was also validated by prior measurements and simulations [95]. Soil properties for the 24 scenario sites were acquired analogously (see supplementary material S5 for detailed soil properties as well as soil classification according to the KA5 classification guideline [91] and WRB [96]). In four cases simulations with the median values of texture classes caused unrealistic soil water simulations in the scenario sites, since the BD and texture class provided by the BÜK200 resulted in a pore volume below the simulated FC according to the internal PTFs. In these cases, the texture was lowered to the lower limit of the respective class.

### 2.3 Management regimes

Ten management regimes representing an intensification gradient from low (1) to high (10) were set up based on management data from long-term survey sites as well as remote sensing data provided by [dataset] [[64], [65], [66]] as model input for scenario simulations. The regimes include management with 1) one to five cuts per year and mineral fertilization (meadows), 2) one to four cuts and grazing, mineral as well as organic fertilization (mown pastures) and 3) grazing only (pasture) (see supplementary material S6 for detailed information of the management regimes). The management operations within each regime were kept constant across all years to allow assessing the effect of different land use practices across changing environmental conditions within each scenario.

The yield levels and fertilizer quantities correspond to the yields of the respective intensity of grassland use as well as their N requirement [63]. The stocking rates of grazing cattle were derived from available management data on grazed grasslands from the validation sites. Therein, the stocking rates as well as grazing duration varied highly. The management regimes account for this variability by reducing the stocking rate under regimes with a longer grazing duration and thus keeping the grazing intensity constant across all regimes. With regard to the N requirement, grazing was considered as an additional cut, in accordance with the fertilizer ordinance [63]. The N content of organic fertilizer was determined based on data provided by the Lower Saxony Chamber of Agriculture [97].

### 2.4 Initialization of scenario simulations

The simulations were carried out for each combination of the 24 selected scenario sites, ten management regimes and the three RCP scenarios with six climate models each. This resulted in 4,320 individual simulations over a period of 110 years (1990–2099), which allows to minimize the uncertainty of future simulations caused by the application of too few scenarios [56]. Due to the conceptual character of the selected scenario sites, it was not possible to initialize them based on the measured SOC content, as was done for the parametrization- and validation sites. Therefore, a uniform initial SOC content of 2 M-% in the topsoil (0–30 cm depth) was assumed, which was the mean value of the validation sites. While a site-specific SOC parametrization is usually more desirable as it produces optimal results, a generalized approach comes with the benefit of the comparability of the individual sites with varying pedogenetic and climatic characteristics [81]. As a result of a sensitivity analysis on CANDY-based simulations [79], it was found that the initial SOC content is the most dominant factor in the first year of simulations and it affects long-term SOC modelling, but its dominance decreases with time, while other site properties increase. To avoid this initialization error and to determine the (model) site specific SOC starting value, simulations were conducted with a 30 year long initialization period, starting simulations in 1990 using ambient climate, following simulations using future climate based on the RCP scenarios from 2020 until 2099. Reference [79] successfully implemented a similar approach with an initialization period of seven years. Due to the relatively slow turnover, SOC changes need to be studied over years and decades, while monthly and daily changes are of relatively minor importance [95]. SOC simulations were therefore conducted in annual time steps and were limited to the topsoil (upper 30 cm), since the majority of vegetation- and land use related effects and nutrient turnover can be observed in this layer [[98], [99], [100], [101]].

### 2.5 Statistical analysis

Statistical analysis was conducted using the open source software R version 4.0.2 and R Studio version March 1, 1073 [102]. Visualization of data was performed with the R package *ggplot2* [103]. Correlation of model input data was explored with the *corrplot* package [104].

#### 2.5.1 Parametrization and validation

To validate the parametrization of the CANDY model, the root mean square error (RSME), relative RMSE (RRMSE) and coefficient of determination (R^2^) were calculated from observed vs predicted SOC contents using the *modeval* function within the *sirad* package [105]. The RMSE, RRMSE and R^2^ are mathematically expressed as follows:

(1) $$RMSE=\sqrt{\frac{\sum_{i=1}^{n}\left(P_{i}-O_{i}\right)²}{n}}$$

(2) $$RRMSE=100\frac{\sqrt{\frac{\sum_{i=1}^{n}\left(P_{i}-O_{i}\right)²}{n}}}{\bar{O}}$$

(3) $$R^{2}=\frac{\sum_{i=1}^{n}\left(P_{i}-O_{i}\right)\cdot \left(O_{i}-\bar{O}\right)}{\sqrt{\sum_{i=1}^{n}\left(P_{i-}\bar{P}\right)²\cdot \sum_{i=1}^{n}\left(O_{i-}\bar{O}\right)²}}$$

with *P* = predicted value, *O* = observed value, $\bar{P}$ = mean of predicted values, $\bar{O}$ = mean of observed values, *n* = number of *P/O* pairs, *i* = individual *P/O* pairs [77,106,107].

#### 2.5.2 Scenario simulations

To analyse the effect of management, soil group and climate on the SOC balance, mixed effects models were fitted using the *nlme* package [108]. For the evaluation of the scenario simulation results, the simulated SOC for each of the 4,320 simulation runs across the simulation period as well as management-, soil- and climate data were aggregated by different means.

The SOC balance of every simulation run was determined as the difference between the last simulated value in the year 2099 and the first simulated value at the beginning of the simulations with the future climate, in the year 2020.

The management regimes were considered as a factor with ten levels, due to a high co-linearity of management operations across the ten management regimes (see also supplementary material S7 for a correlation matrix of the management data).

Soil data were aggregated into four levels by their texture class into sands, silts, clays and loams, in accordance to the KA5 soil mapping guideline [91] (see also supplementary material S5). This enables a practical interpretation of the simulation results.

Climate data were taken into account both as qualitative predictors (RCP scenario (three levels) and its projections (six levels) with a total of 18 levels), and as quantitative predictors, in form of site-specific long-term (2020–2099) means. These were calculated in three ways: 1) as averages of annual means (air temperature and global radiation) and of annual sums (precipitation) (annual average means – AAM), 2) as the average standard deviation within all years (mean intra-annual variability – MIV) and 3) as the variance of annual means (air temperature and global radiation) and annual sums (precipitation) across the entire simulation period (inter-annual variance of means – IVM). The AAM is a relatively static descriptor of climatic conditions, the MIV in turn expresses how much the air temperature, precipitation and global radiation fluctuate due to seasonality, while the IVM describes how much the annual means/sum change over the long-term (80 years). The aggregation by three different methods was motivated by studies reporting that the magnitude of absolute climate metrics may be second to the effects of seasonal fluctuation and long-term change [109,110] as well as a requirement to investigate the impact of seasonality, particularly growing season precipitation, on SOC sequestration [46].

#### 2.5.3 Model inference

A multimodel inference approach was applied [58], which allows to compare and rank several models representing competing hypotheses using information criteria such as the Akaike information criterion (AIC; [111]). At first four global models (GMs) with the fixed effects management (10 levels), soil group (4 levels), climate (see below) and two- and three-way interactions and the random effect site (24 levels) were fitted. The structure of all four GMs can be summarized as follows:

GM=SOCbalance∼(management+soilgroup+climate)^3+randomeffect

The explanatory variable climate was included either as qualitative predictors (RCP scenario (3 levels) and its projections (6 levels), with a total of 18 levels, GM-SP), or as quantitative predictors air temperature, precipitation and global radiation aggregated by the same method (GM-AAM, GM-MIV, GM-IVM). Interactions between climate variables were not included into the models due to high complexity (see also supplementary material S8 for a correlation matrix of climate data). The variance inflation factor (VIF) was calculated for these models and resulted in values < 2, attesting minor co-linearity between explanatory variables.

Subsequently, candidate models (CMs) were fitted for each of the four GMs, which included all possible combinations of predictors and their interactions using the *dredge* function of the *MuMIn* package [112]. The best CMs within dAIC<2 and non-spatially correlated residuals were interpreted using the *effects* package to produce confidence intervals [113], and the *emmeans* package [114] and the *multcomp* package [115] to perform the post hoc test at an alpha of 0.05. Adjusted R^2^ are reported for the model in total, for the fixed effects as well as for the random effects using the *rsq* package [116].

The model performance was assessed by plotting the residuals and several adjustments were made: A variance structure was incorporated into the GMs to account for different heterogeneity between soil type groups using the *varIdent* function of the *nlme* package [108,117]. This variance function improved the fit of models considerably (see supplementary material S9 and S10 for residuals plots).

The spatial distribution of sites was taken into account by fitting spatial correlation structures (none, Gaussian, spherical, rational, exponential, linear) in the GMs using the site coordinates and comparing them by means of the AIC. For this purpose, the site coordinates were modified by a small random number in order to avoid problems related to null-distances due to the usage of multiple climate- and management scenarios per site (see also supplementary material S11 for plotted residuals in dependence of latitude and longitude). Furthermore, spatial autocorrelation (SAC) in residuals was tested for the best CMs at the site-level using the Moran's I at p < 0.05 and the *ape* package [118].

## 3 Results

### 3.1 Parametrization and validation

**Fig. 2:**
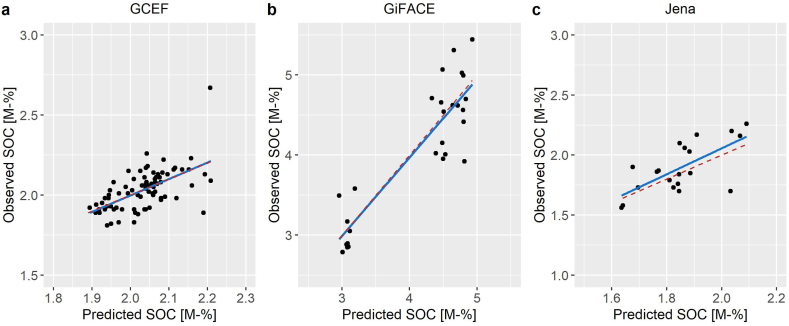
Observed and predicted SOC contents in the topsoil layers of the parametrization dataset. Continuous line shows the fitted linear regression, dashed line shows the theoretical ideal 1:1 fit.

**Fig. 3:**
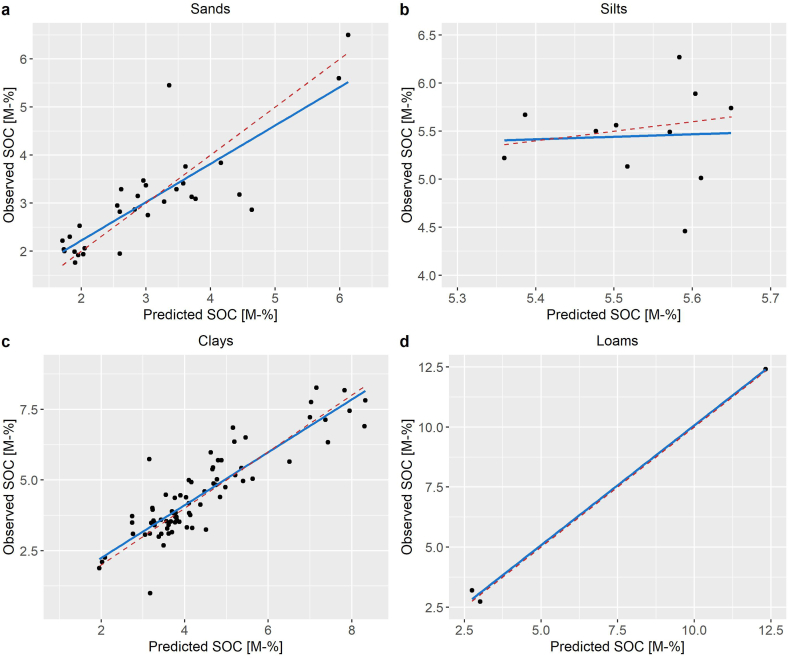
Observed and predicted SOC contents in the topsoil of the long-term soil survey sites in Germany by soil group. Continuous line shows the fitted linear regression, dashed line shows the theoretical ideal 1:1 fit.

In comparison to the initial CANDY parametrization, the transpiration coefficient was increased from 0.8 to 8.0 kg mm^−1^. In all cases, the re-parametrization resulted in an increase of the performance in comparison to the initial version. The duration of the vegetation period displayed a minor effect on the performance statistics. The overall best performance was achieved by increasing the duration of the vegetation period from initially 254 to 256 days. The model performance across all parametrization data displayed a good fit with an RRMSE of 8.22% and R^2^ of 0.955 (Table 1, Fig. 2). The new parametrization was validated by 23 long-term soil survey sites. Despite a poor R^2^ on silts, likely due to relatively low SOC spread, the data produced an overall RRMSE of 15.99% an R^2^ of 0.845, confirming a reliable parametrization across all sites (Table 2, Fig. 3).

**Table 1:** Model performance statistics after complete parametrization of the CANDY model based on the experimental sites of the GCEF-, GiFACE- and Jena-datasets, including number of available SOC measurements (n).

Dataset	RMSE [M-% SOC]	RRMSE [%]	R^2^	n
GCEF, Bad Lauchstädt	0.101	4.96	0.359	80
GiFACE, Giessen	0.377	9.22	0.793	27
Jena Experiment, Jena	0.155	8.21	0.472	20
**All data**	0.200	8.22	0.955	127

**Table 2:** Model performance statistics for the validation sites in Lower Saxony, Brandenburg, Baden-Württemberg and Thuringia, grouped by soil type and including number of available SOC measurements (n).

Soil type	RMSE [M-% SOC]	RRMSE [%]	R^2^	n
Sand	0.650	21.31	0.692	31
Silt	0.473	8.68	0.002	11
Clay	0.723	16.16	0.784	76
Loam	0.312	5.10	0.995	3
**All data**	0.677	15.99	0.845	121

### 3.2 Scenario simulations

**Fig. 4:**
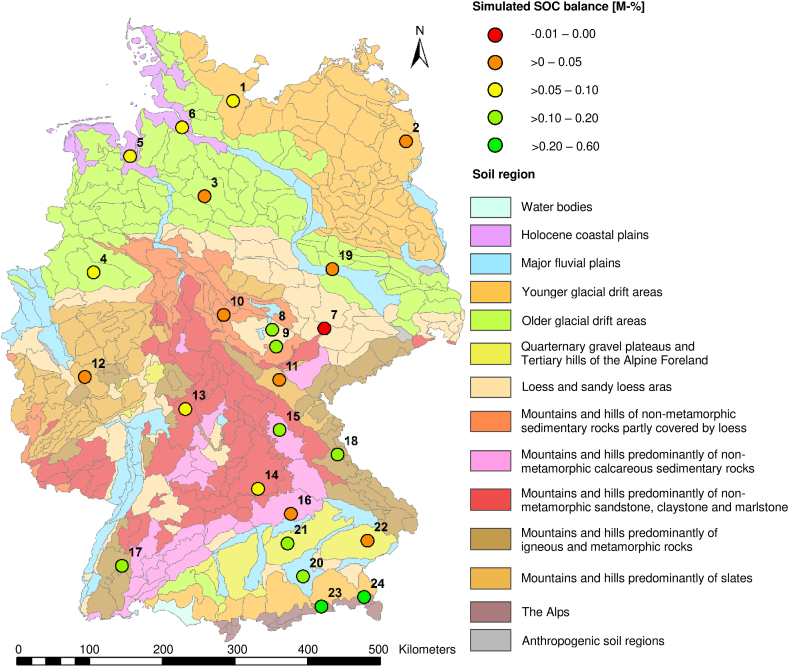
Simulated SOC balance (averaged across three RCP scenarios, six projections and ten management regimes) for the 24 scenario sites. Soil regions and natural landscapes in accordance to Ref. [67] and [dataset] [59].

Fig. 4 shows the simulated SOC balance for the 24 scenario sites with averages of three RCP scenarios, six projections and ten management regimes. The only site with an overall negative mean SOC balance (a clay-rich soil on site 7, see also supplementary material S5) was located in the soil region “loess and sandy loess area” in Thuringia. This site displayed low precipitation amounts (<620 mm) and moderate to high mean air temperatures (10.30–11.63 °C) across all three RCP scenarios (see also supplementary material S3). The majority of sites with a strong SOC increase were located in the Alps regions in southern Germany, particularly Bavaria (sites 20–24). These fine-textured sites (30–45 M-% silt and 20–40 M-% clay content in the topsoil) were characterized by high precipitation amounts (partially above 1.000 mm) and low mean air temperatures (<10.0 °C for RCP 2.6 and 4.5). Sites in northern Germany (sites 1, 5 and 6) displayed in average only minor increases as a result of moderate precipitation (780–840 mm across all scenarios) and air temperature (9.48–11.35 °C across all scenarios).

#### 3.2.1 SOC-balance in relation to climate, soil and management

The multimodel inference identified site properties (climate, management and soil type) and interactions that substantially affect the simulated SOC balance. The best fit for the GM-AAM and GM-MIV models was achieved with an exponential and Gaussian structure respectively, while the GM-IVM model produced the best fit using no spatial correlation structure. In all cases, only one CM was within dAIC<2 (see Table 3). Among the models which use qualitative climate predictors (projection and scenario, GM-SP), the best CM included all terms of the GM except for the interaction between soil group and climate scenario and all three-way interactions. Among the AAM and MIV models respectively, the best CMs were the GMs. The best CMs, CM-AAM-1 and CM-MIV-1, therefore solely differed by their aggregation of climate data and SAC structure, with the same predictor terms and interactions (but different coefficients). Among the IVM models, the best CM included all terms of the GM except for the three-way interaction between soil group, management and the IVM of temperature. From all GMs, GM-AAM displayed the highest empirical support with the lowest AIC, while GM-SP displayed the lowest, with the exception of the null model M0.

**Table 3:** Summary statistics of the global models (GMs) and null model (M0) in comparison to the best fitting candidate models (CMs) based on a dAIC<2. sg = soil group, mng = management regime, scn = scenario, prj = projection, pre = precipitation, temp = air temperature, rad = global radiation. The models are grouped by the method by which climate data were aggregated (SP, AAM, MIV, IVM). The dAIC always refers to the best CM within the climate aggregation groups.

Model	Explanatory variables	df	AIC	dAIC	R^2^		
					total	fixed	random
GM-SP CM-SP-1	(sg + mng + scn + prj)^3 sg + mng + scen + prj +sg:mng + sg:prj +scen:mng + scen:prj +mng:prj	455 140	−9162.7 −9689.4	526.7 0.00	0.94 0.95	0.52 0.57	0.42 0.38
GM-AAM CM-AAM-1	sg*mng*(pre + temp + rad) sg*mng*(pre + temp + rad)	166	−13145.0	0	0.97	0.66	0.31
GM-MIV CM-MIV-1	sg*mng*(pre + temp + rad) sg*mng*(pre + temp + rad)	166	−12772.2	0	0.97	0.83	0.13
GM-IVM CM-IVM-1	sg*mng*(pre + temp + rad) sg*mng*(pre + rad)+temp +mng:temp + sg:temp	165 138	−11904.8 −11913.2	8.4 0	0.96 0.96	0.68 0.68	0.28 0.28
M0	–	6	−2332.6	–	0.28	0.00	0.28

Among the SP, AAM, MIV and IVM models, all models displayed a similar overall performance, judged by the total R^2^ and AIC, and thus outperform M0. The proportion of variation explained by the fixed effects was highest for the MIV models, and similar for the AAM and IVM models. Conversely, the proportion of variation explained by the random effects was lowest for the MIV models.

SAC within residuals persisted for the models CM-AAM-1, CM-IVM-1 and CM-SP-1 at p < 0.05, while no SAC in residuals could be detected for CM-MIV-1 at p > 0.05 (see Table 4). From all available models, only CM-MIV-1 fulfilled the requirements for model interpretation of having a dAIC<2 and non-spatially correlated residuals. Its suitability was supported by the higher R^2^ for fixed effects.

**Table 4:** Observed and predicted Moran's I values for the best candidate models. Significant spatial autocorrelation persists at p < 0.05.

Model	CM-AAM-1	CM-MIV-1	CM-IVM-1	CM-SP-1
observed	0.062	−0.036	0.055	0.032
predicted	−0.043	−0.043	−0.043	−0.043
sd	0.035	0.043	0.036	0.028
p	0.003	0.868	0.006	0.008

#### 3.2.2 Interpretation of effects

**Fig. 5:**
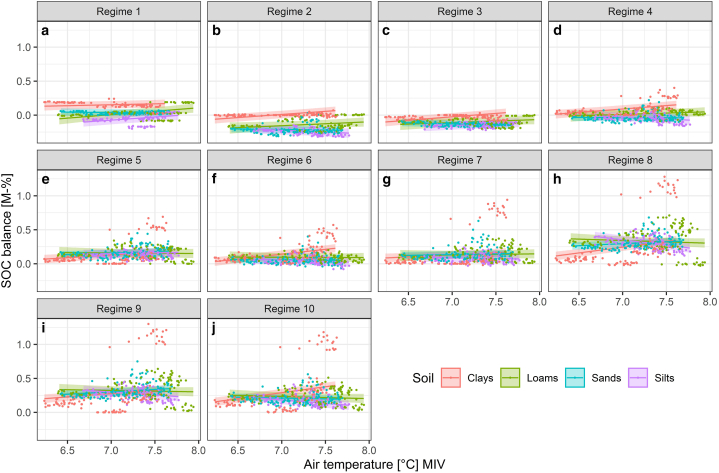
Linear regressions (lines) and 95% confidence intervals (ribbons) of the effect of the mean intra-annual variability (MIV) of temperature on the SOC balance [M-%] in dependence of the soil type and management regime according to the CM-MIV-1 model.

Based on the model CM-MIV-1, changes in the MIV of temperature exercised minor effects on the SOC balance at lower to moderate management intensity (Fig. 5, regimes 1–5). Under an extensive grazing regime (regime 1) with increasing MIV of temperature, the SOC balance of clays remained fairly stable, with an increase by 0.027 M-% per °C (see also supplementary material S12 for the SOC change per one unit increase of air temperature, precipitation and global radiation). Loams and silts increased by 0.101 and 0.091 M-% per °C respectively, while sands decreased by 0.013 M-% per °C. Differences between management regimes and soil groups became more pronounced at higher management intensity. At five cuts (regime 10), the SOC balance of clays increased by 0.176 M-% per °C, the balance of loams, sands and silts decreased by 0.032, 0.024 and 0.102 M-% per °C respectively.

**Fig. 6:**
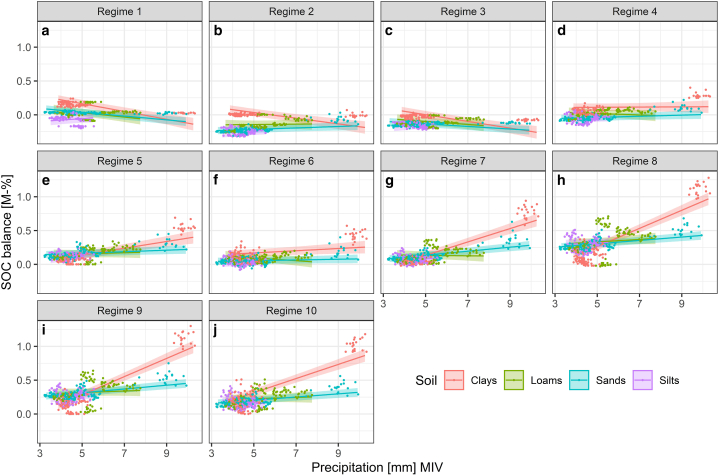
Linear regressions (lines) and 95% confidence intervals (ribbons) of the effect of the mean intra-annual variability (MIV) of precipitation on the SOC balance [M-%] in dependence of the soil type and management regime according to the CM-MIV-1 model.

An increase in the MIV of precipitation resulted in a decreasing SOC balance at lower management intensity (Fig. 6, regimes 1–3) and an increasing SOC balance at higher management intensity (regimes 7–10). Under extensive grazing management (regime 1), the SOC balance decreased by 0.058 M-% per mm on clays, by 0.033 M-% per mm on loams, and by 0.028 M-% per mm on sands. Silts were the only soil group experiencing a slight increase by 0.021 M-% per mm. Under intensive management with five cuts (regime 10), clays showed the highest increase, by 0.105 M-% per mm, followed by sands increasing by 0.024 M-% per mm and loams increasing by 0.009 M-% per mm. The SOC balance of silts in turn decreases by 0.022 M-% per mm.

**Fig. 7:**
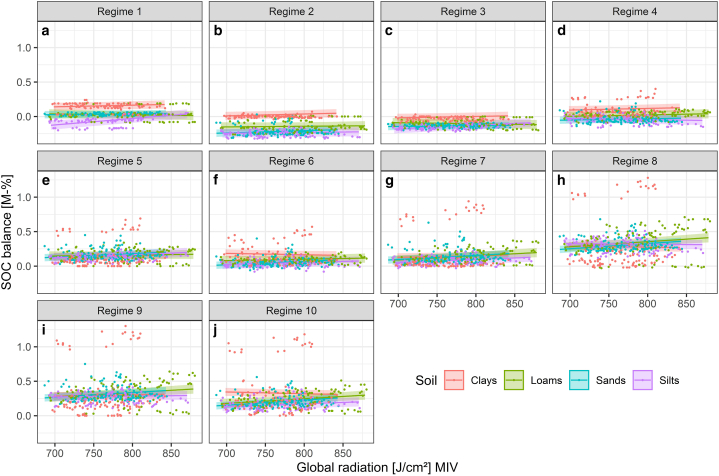
Linear regressions (lines) and 95% confidence intervals (ribbons) of the effect of the mean intra-annual variability (MIV) of global radiation on the SOC balance [M-%] in dependence of the soil type and management regime according to the CM-MIV-1 model.

Changes due to increasing MIV of global radiation displayed similar effects across all management regimes (Fig. 7). Under an extensive grazing regime (regime 1) and increasing MIV of global radiation, the SOC balance increased by 0.024 M-% per 100 J cm^−2^ on clays, by 0.010 M-% per 100 J cm^−2^ on sands and by 0.093 M-% per 100 J cm^−2^ on silts, while it decreased by 0.012 M-% per 100 J cm^−2^ on loams. Under a five-cut regime (regime 10) with increasing MIV of global radiation, the SOC balance increased by 0.067 M-% per 100 J cm^−2^ on loams, by 0.073 M-% per 100 J cm^−2^ on sands and by 0.040 M-% per 100 J cm^−2^ on silts, while it decreased by 0.025 M-% per 100 J cm^−2^ on clays.

**Fig. 8a:**
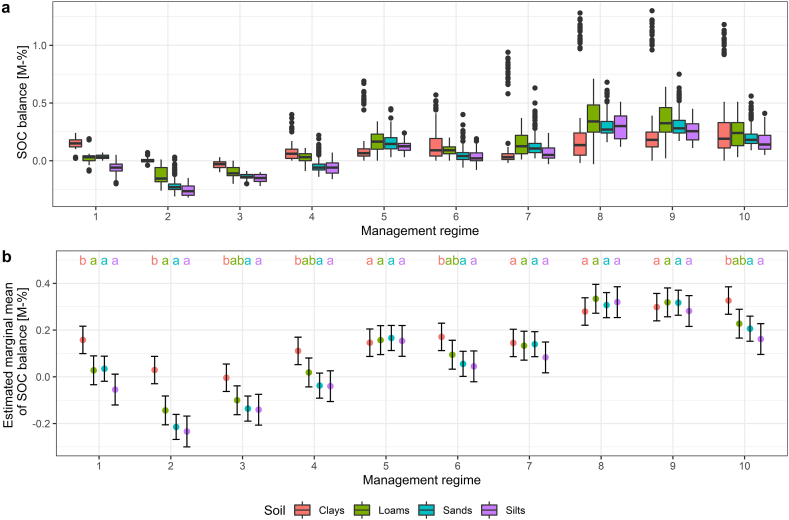
Simulated SOC balance [M-%] in the topsoil (0–30 cm) for the period 2020–2099 across 24 sites, grouped by soil group and management regimes. Fig. 8b: Estimated marginal means and 95% confidence intervals (error bars) obtained from the CM-MIV-1 model for the SOC balance, grouped by management regime and soil type. Post hoc test was applied within each management regime. Soil groups that do not share a letter differ significantly (p < 0.05).

Regardless of soil group, the highest mean SOC increase and simultaneously highest variability during the simulation period was simulated for the regimes 8 (+0.31 ± 0.23 M-%) and 9 (+0.30 ± 0.22 M-%), which represent a land use with four cuts, with regime 9 additionally including a grazing period (Fig. 8a). An increase to five cuts (management regime 10) resulted in a lower SOC increase across all sites (+0.23 ± 0.20 M-%) in comparison to a four-cut use. SOC losses were determined under management regimes 2 (−0.15 ± 0.11 M-%) and 3 (−0.11 ± 0.06 M-%), which represent a meadow and mown pasture respectively, both under a single cut regime. A low to moderate increase in SOC balance was determined under regimes 1 (+0.04 ± 0.09 M-%), 4 (+0.01 ± 0.09 M-%), 5 (+0.15 ± 0.12 M-%), 6 (+0.08 ± 0.10 M-%) and 7 (+0.13 ± 0.17 M-%). Overall, according to estimated marginal means provided by CM-MIV-1 (Fig. 8b), clays displayed either a similar or a significantly higher SOC balance than loams, sands and silts under all management regimes. Silts in turn displayed the overall lowest, though not significantly lower than sands and loams, SOC balance across all management regimes.

## 4 Discussion

In this study, the SOC balance obtained from the CANDY model was analyzed in relation to the effects of management intensity, soil type and climate using 24 representative grassland sites. Management was identified as the most important driver affecting SOC sequestration, though different responses of soil types to the effects of climate were detected. Among climatic properties, the precipitation during growing season exerted the highest impact on the SOC balance.

### 4.1 Model parametrization and validation

The CANDY model performed well in predicting the SOC content, as shown by the error statistics for the parametrization and validation data. While the R^2^ was fairly small (<0.5) in the GCEF and Jena parametrization datasets, likely due to a high variability of SOC measurements (see e.g. Refs. [78,119]) and a relatively short period of available measurements, for all data a value of >0.9 was achieved, attesting to a high reliability across wide pedoclimatic conditions and land use regimes. The RMSE was overall within a good range on the parametrization sites. Reference [81] reported similar RRMSEs (5.4–10.5%) for CANDY-based SOC simulations in Austria. The RMSEs of this study were also in accordance with values reported for prior CANDY-based simulations with the GCEF-dataset conducted by Refs. [74,76]. Therein, the RMSE ranged between 0.05 and 0.15 M-% SOC.

The R^2^ for the validation sites confirmed the reliability of long-term simulations on a variety of climates, soil- and management types across Germany, despite RRMSEs up to 22% for sandy soils in the validation data and despite the number of available sites and years of data across the three validation datasets varying highly (see data availability in the material and methods section). A high spatial and temporal variability in SOC measurements is well known to result in a relatively high error [31,120,121], particularly when considering the high fluctuations of measured SOC contents and the necessity to complete missing management-, soil- and climate data from third party sources on the validation sites. The single low R^2^ on silts was likely a result of the relatively small range of measured SOC contents on the only two available sites in the dataset. Considering the overall good performance of the validation dataset (R^2^ > 0.8), the CANDY model was considered reliable for the evaluation of long-term future climate scenarios.

### 4.2 Scenario simulations

#### 4.2.1 Importance of seasonal fluctuations

The multimodel inference approach and the investigation of spatial autocorrelation substantiated the importance of seasonal fluctuations of climate metrics (precipitation, air temperature, global radiation): The mean intra-annual variability (MIV), which reflects on the seasonality, was the most reliable aggregation method for the prediction of the SOC balance. Using annual sums and means (AAM) produced a higher empirical support judged solely by the AIC. However, only the MIV model successfully accounted for spatial autocorrelation, which was a central issue detected in our data, and furthermore displayed the best performance, as per the higher fixed R^2^ and lower random R^2^.

Changes of the seasonal weather regimes were therefore more relevant to the SOC sequestration than the mere increase or decrease of the annual mean of temperature and annual sum of precipitation [82]. Several studies also report the prevalence of seasonal effects and changes in temperature or precipitation regimes over the effects of uniform changes [43,110,[122], [123], [124], [125]]. Particularly unusually long periods between rainfall events result in water stress, thus decreasing plant productivity and sequestration potential [125]. An increasing MIV coincides with higher seasonal amplitudes, and may therefore indicate stress caused by dryness, wetness, heat or cold [19,82]. However, in this study, precipitation aggregated by AAM and MIV was highly correlated (see supplementary material S8). This implies an increasing seasonality of precipitation with an increasing annual sum of rainfall. All but one site showed increasing precipitation under the RCP 2.6 scenario, with even higher increases under the RCP 8.5 scenario, in comparison to ambient climate (see supplementary material S1 and S3). Consequently, an increasing MIV of precipitation did not result in dry periods in our dataset, since in large parts of Germany the majority of rainfall occurs during the growing season (see e.g. Refs. [82,126]). Summer dryness is thus more likely to occur in regions in Germany with a low seasonality of precipitation. The relative importance of the variability of precipitation regimes may furthermore differ on wet, intermediate and dry sites [125]. In our study, the seasonal distribution of rainfall events was most impacting on sites experiencing excessive dryness. A low MIV of precipitation, and therefore its seasonality, by proxy indicates a risk of decreasing SOC. Accordingly, a simulation study by Ref. [49] using the PaSim model revealed that the net primary production was promoted by increased precipitation particularly at dry sites. Reference [124] also reason that the distribution and magnitude of rainfall events play a dominant role for the recovery of water contents in deeper horizons primarily in arid and semi-arid regions. Within the scope of this study however, it was not further possible to identify locations-specific differences regarding the relative importance of either MIV or AAM on the sequestration potential, which presents a future research topic.

#### 4.2.2 Impact of changing precipitation and air temperature

Our results showed the highest climate-related variability of SOC sequestration due to changes in precipitation, whereas air temperature and particularly global radiation exerted a smaller impact. According to Ref. [19] soil moisture was the main factor governing sequestration in German soils. A dominance of mean precipitation over mean temperature on turnover rates was also documented by Ref. [10], with sequestration increasing from arid and cold to warm and humid climates. Hence, in our study the highest sequestration potential was ascertained in the southern region of Germany, which coincided with the highest precipitation rates (see also Fig. 4 and supplementary material S1 and S3). Similarly, reference [44] detected the highest sequestration rates in Europe in the alpine region, based on simulations with the ORCHIDEE-GM model. A review by Ref. [26] attributed correspondingly lower SOC accumulation rates to arid climates, and reference [127] reported that increasing altitude, and therewith precipitation, coincided with higher SOC contents. A similar effect was demonstrated by Ref. [128], who showed that topsoil SOC stocks increase with elevation and rainfall amounts. Nevertheless, increasing air temperature with future climate change may promote SOC sequestration [31]. In accord with our results, reference [129] conclude that temperate grasslands would likely become C sinks with future climate change as a result of increasing precipitation and temperature. Grasslands in Germany are predominantly situated in the alpine and low mountain regions and will therefore benefit from increasing precipitation and temperature: As an effect of a specific warming level above 1 °C in comparison to pre-industrial climate (1881–1910) reference [3] presented evidence suggesting that most German grassland soils would experience an increased net biome productivity and a longer growing season, presenting potential for SOC sequestration, though increasing temperature may result in increasing microbial activity, higher mineralization rates and therefore SOC losses [127]. At warming levels reaching 3.5 °C reference [3] reported that increasing droughts would shorten the vegetation period, turning former C sinks to sources. Favourable effects of increasing temperature are counteracted by reduced precipitation particularly in the summer months [82]. This applies in particular for sites with already low summer precipitation. Positive feedbacks on the radiative forcing caused by anthropogenic climate change may further limit the terrestrial sequestration potential [130]. Reduced vegetation cover changes the surface albedo, causing higher amounts of radiation hitting the soil surface and increasing soil temperature, exacerbating already present dryness stress [82].

#### 4.2.3 Interaction of climate and management practices

In our simulations, the effect of climate change on the SOC balance was largely driven by management practices. A prevalence of the effect of land use over the effect of climate was determined both by measurements [19] and by long-term scenario simulations [43,50] and confirmed in a recent review [15]. However, as our simulations illustrated, due to the complex interaction of management and precipitation, simulation-based assessments are required to include a multitude of management- and climate regimes, as it is not possible to deduce ubiquitous rules with only one or few land use regimes. Reference [49] for instance found temperature and precipitation dominated the effect of management, which is in strong contrast to our results. In our study, management pronounced the climate-related differences in SOC sequestration between different sites. The study by Ref. [49] however included only two management regimes (cutting and grazing) on three sites with moderate to high precipitation (625, 995, 1033 mm), which simply may not suffice to explore the effect of varying management intensity, as illustrated by our results. Since the management regimes used in our study were created artificially and cover a wide spectrum from extensive to intensive management, based on information from long-term survey sites, the German fertilizer ordinance [63] and remote sensing data [dataset] [[64], [65], [66]], uncertainties largely stem from the application of future climate datasets. Reference [56] further substantiated this, arguing that high uncertainties may arise as an effect of simulations with a single climate scenario. Our study accounted for this by the application of three contrasting climate scenarios, each realized by six different climate projections [57,[68], [69], [70], [71], [93]]. As evident by the relatively small effect sizes of changing climate metrics in comparison to the effects of management (see Fig. 5, Fig. 6, Fig. 7) as well as small differences between climate projections (i.e. climate models that realize a given scenario) within each scenario (see supplementary material S12 and S13), the uncertainty associated with predicting the impacts of future climate scenarios is small. We therefore consider our results robust to the assumptions underlying our simulations (i.e. future climate change). As a caveat though, the applied RCP scenarios merely quantify the future radiative forcing and the resulting change in air temperature, precipitation and global radiation [57]. They represent a best case, worst case and middle of the road scenario of the efforts undertaken to mitigate future climate change. The exact emissions and therefrom resulting changes in the radiative forcing, however, are subject to various and complex socio-economic and political processes. These processes in turn need to be explored with the aid of the recent SSP scenarios [92] in order to guide decision making in policies aiming to mitigate climate change.

With increasing management intensity (up to five cuts), all scenario sites became C sinks on average. Grassland management practices that increase yield, such as increasing cutting frequency, organic and mineral fertilization and grazing intensification, generally also promote SOC sequestration by vegetation growth and rhizodeposition [[29], [30], [31]], thus explaining higher sequestration rates under a four- and five-cut regime in our simulations. The beneficial effect of a higher management intensity prevails at increasing precipitation, since additional vegetation regrowth due to cutting requires additional water input. Considering precipitation increases as an effect of climate change in our simulation sites (see supplementary material S3), a moderate intensification of extensively used grassland, as investigated in our scenario simulations, is an adequate method to increase SOC sequestration [21,31,32]. An extensive use with two or less cuts per year (regimes 1 to 4) in turn is detrimental to long-term sequestration. Nevertheless, an increasing cutting frequency typically requires additional N-fertilizer input and more machine traffic, which may result in higher GHG emissions [3,44]. The production and application of mineral N fertilizer furthermore causes high CO_2_ emissions, requiring life cycle assessment (e.g. Ref. [131]) in order to gauge the benefits and risks. Increasing SOC sequestration at the cost of increased N emissions is highly questionable, as N_2_O, which is a by-product of denitrification, has the 296-fold global warming potential of CO_2_ over a period of 100 years and is therefore a high impact for global warming [4,132,133]. Intensification of land use may furthermore be in conflict with the preservation of biodiversity [115,[134], [135], [136], [137]], requiring a thorough consideration of a landscapes potential for achieving the respective goals at the farm scale.

#### 4.2.4 Varying soil type related responses to climate and management

According to the meta-analysis by Ref. [129] and the review by Ref. [138], different soil types respond differently to the effects of climate and management. But in our study sands, silts and loams displayed a similar response to management on average, since the differences between the three soil types under the same management were not significant. Clays in turn showed the highest SOC gains on average. According to the CANDY-simulations, clays in Germany may accumulate up to 1 M-% SOC across the period of 80 years under four cuts (regimes 8 and 9) annually. The effects of land use generally predominate pedogenetic properties [27]. In general, to allow carbon sequestration enough mineral surface is required [139]. This limits the potential of coarse soils even under optimal management and explains higher SOC gains in clay soils. An increase of the cutting frequency at increasing MIV of temperature resulted in a lower SOC balance on most soil types except clays. Differences due to temperature between soil types furthermore largely persisted at higher management intensity (four to five cuts), with silts being most susceptible to temperature increases.

However, our analysis also revealed that the potential for SOC sequestration of clays can be negated under certain conditions: With an increasing MIV of precipitation, i.e. higher summer precipitation, clays displayed the highest potential for both C losses and gains, depending on management. A positive SOC balance was achieved with two or more cuts annually, while extensification resulted in losses. Under increasing precipitation, clays are therefore high-risk/high-gain-sites. An interpretation of the effect of precipitation on silts is hardly viable due to their prevalence in dry regions, therefore occupying only a small spectrum of the overall investigated precipitation pattern. However, according to the estimated marginal means, silts will gain SOC as a result of increasing precipitation with future climate change. The relatively minor changes due to changing precipitation patterns across different management regimes on loams and sands imply a lower response to climate change effects, though increasing the number of cuts also promoted sequestration with higher precipitation on these two soil types, albeit to a lesser extent than on clays. The smaller apparent effect is likely due to high infiltration rates and relatively low water storage capacity [139,140], which reduces the beneficial effect of higher precipitation. Although to a lesser extent, sandy soils benefited from increasing precipitation under future climate in our simulations. However, sands do not constitute typical grassland soils due to their overall low water storage abilities.

## 5 Conclusion

Using a broad dataset consisting of 24 representative grassland sites across Germany, ten management regimes applied to all sites, and 18 site-specific future climate datasets, we investigated the combined effects these properties exert on the future SOC sequestration potential of grasslands. Our results showed that changes in seasonal weather patterns were a more reliable predictor for the SOC sequestration than their absolute means. Precipitation, which increased predominantly at all sites and in all climate scenarios, was more relevant to the SOC sequestration in our survey region than temperature or global radiation. However, in contrast to many reports, an increasing seasonality of rainfall (i.e. higher MIV) did not reduce SOC in our dataset, since the majority of rainfall in Germany occurs during the growing season. Consequently, increasing seasonality promoted SOC sequestration.

The effect of management was dominant over the effect soil type and amplified climate-driven differences between soils. Averaged across all climate scenarios, under an extensive use (two or fewer cuts) all four soil types (sands, silts, clays, loams) experienced minor changes or even SOC losses. At higher management intensities (four to five cuts per year) all soil types increased in SOC. A moderate intensification (three or more cuts, with an optimum at four cuts) therefore promoted sequestration in our representative sites, though the gain of increased sequestration at a higher risk of N emissions due to higher required fertilization rates must be carefully considered. Overall, clay soils displayed the highest potential for long-term SOC accumulation across all climate regimes and land uses. However, under extensive use (two or fewer cuts) in combination of increasing precipitation, the potential for SOC losses was also highest on clays as a result of SOC mineralization surpassing sequestration. Loams and sands in turn displayed the least changes due to varying seasonality of precipitation under all management regimes as an effect of a low particle surface limiting sequestration. Increasing management intensity promoted SOC sequestration particularly at higher precipitation. In our dataset, the highest sequestration rates were thus determined in Southern Germany, which coincides with the highest precipitation amounts. Our study showed that substantial SOC gains can be expected under grassland under adequate management over the next decades. However, due to the long time until SOC changes are measurable, management practices that aim at fostering SOC sequestration need to be implemented early in order to achieve the desired sequestration effect.

For future studies scenario simulations should include a multitude of management regimes, since the effect that climate, particularly precipitation, exerts on SOC varies highly with management intensity. Simulations including management regimes that merely represent two extremes may be insufficient to detect those interdependencies. In conclusion, particularly decreasing summer precipitation is a central factor threatening future SOC sequestration, though the majority of the representative grassland sites selected for our study will experience increasing summer precipitation. Lower summer rainfall can partly be counteracted by management practices, though there is a demand to quantify the locations-specific relative importance of seasonality and absolute means of climate metrics on the sequestration potential. As a result of increasing seasonality of precipitation, grassland management is recommended on the representative sites under future climate change, since 1) the permanent vegetation provides a protective cover against erosion, 2) increasing precipitation amounts aid the recovery of soil water and 3) grassland management with three to five cuts annually contributes to the mitigation of climate change via increased SOC sequestration.

## Author contribution statement

Matthias Robert Filipiak: Conceived and designed the experiments; Performed the experiments; Analyzed and interpreted the data; Contributed reagents, materials, analysis tools or data; Wrote the paper.

Doreen Gabriel: Analyzed and interpreted the data; Contributed reagents, materials, analysis tools or data; Wrote the paper.

Katrin Kuka: Conceived and designed the experiments; Analyzed and interpreted the data; Contributed reagents, materials, analysis tools or data; Wrote the paper.

## Data availability statement

Data included in article/supplementary material/referenced in article.

**Funding** **statement**

The project was funded by the Federal Ministry of Food and Agriculture (BMEL) [grant number 2818300916]. The research project is assigned to the guideline on the funding of innovations for sustainable grassland management in the programme for the promotion of innovation of the Federal Ministry of Food and Agriculture (BMEL). It was carried out by the Federal Agency for Agriculture and Food (BLE) within the framework of the Innovation Promotion Programme.

## Declaration of competing interest

The authors declare that they have no known competing financial interests or personal relationships that could have appeared to influence the work reported in this paper.
