# Supplementary material for: Simulation-based assessment of the soil organic carbon sequestration in grasslands in relation to management and climate change scenarios
Source: Heliyon. 2023 Jun 17;9(6):e17287. doi: 10.1016/j.heliyon.2023.e17287 (PMC10333473; doi:10.1016/j.heliyon.2023.e17287)
Supplement: Multimedia component 1 [file mmc1.docx]

Supplementary material to:

Filipiak, M., Gabriel, D., Kuka, K (2022): Simulation-based assessment of the soil organic carbon sequestration in grasslands in relation to management and climate change scenarios.

S1: Ambient (1990 – 2019) climate data for the 24 scenario sites as per Fig. 4, including climate station name and number as well as coordinates of the scenario site. Displayed climate data are: Mean air temperature in 2 m (Temp.), mean annual precipitation sum (Prec.) and mean daily sunshine duration (Sun.). The data was provided by the DWD in Brunswick.

| **Site nr.** | **Climate data** | | | **Station number and name** | **Coordinates (ETRS 89 Zone 32 N)** | |
| --- | --- | --- | --- | --- | --- | --- |
|  | **Temp. [°C]** | **Prec. [mm]** | **Sun. [h]** |  | **Lat** | **Long** |
| 1 | 9.47 | 633.48 | 4.59 | 4625 Schwerin | 5981232.791 | 600632.6635 |
| 2 | 9.38 | 525.12 | 4.75 | 1869 Grünow | 5925692.302 | 838739.8694 |
| 3 | 9.40 | 783.15 | 4.24 | 4745 Soltau | 5850022.054 | 561354.6638 |
| 4 | 10.30 | 739.44 | 4.38 | 1766 Münster/Osnabrück | 5745383.778 | 408576.8236 |
| 5 | 9.88 | 676.08 | 4.36 | 691 Bremen | 5905259.737 | 458924.1372 |
| 6 | 9.76 | 781.45 | 4.40 | 1975 Hamburg-Fuhlsbüttel | 5944929.404 | 530520.4968 |
| 7 | 10.17 | 615.16 | 4.65 | 2928 Leipzig-Holzhausen | 5668108.189 | 726361.7216 |
| 8 | 9.75 | 483.97 | 4.54 | 198 Artern | 5666391.157 | 654474.1205 |
| 9 | 10.30 | 592.58 | 4.53 | 5424 Weimar-Schöndorf | 5643160.975 | 659905.5265 |
| 10 | 8.66 | 704.22 | 4.34 | 2925 Leinefelde | 5686619.661 | 587667.7663 |
| 11 | 7.70 | 713.32 | 4.42 | 2261 Hof | 5597247.988 | 664123.618 |
| 12 | 10.71 | 802.16 | 4.33 | 2667 Köln-Bonn | 5601235.436 | 396621.051 |
| 13 | 9.07 | 806.80 | 4.60 | 3287 Michelstadt-Vielbrunn | 5557178.958 | 534909.6763 |
| 14 | 9.49 | 667.96 | 4.79 | 5440 Weißenburg-Emetzheim | 5447302.341 | 635079.6951 |
| 15 | 9.58 | 637.51 | 4.56 | 282 Bamberg | 5528471.917 | 664595.09 |
| 16 | 9.10 | 692.00 | 4.83 | 2700 Kösching | 5412865.008 | 680353.7853 |
| 17 | 11.22 | 822.53 | 4.99 | 2812 Lahr | 5341255.83 | 447780.0085 |
| 18 | 8.68 | 598.28 | 4.44 | 5397 Weiden | 5494562.014 | 744560.497 |
| 19 | 9.92 | 563.34 | 4.73 | 5629 Wittenberg | 5749759.486 | 737313.01 |
| 20 | 10.13 | 941.71 | 5.02 | 3379 München-Stadt | 5326602.602 | 696929.7147 |
| 21 | 8.94 | 752.34 | 4.82 | 232 Augsburg | 5372124.357 | 675712.8542 |
| 22 | 9.34 | 781.59 | 4.69 | 73 Aldersbach-Kriestorf | 5375855.28 | 785682.9702 |
| 23 | 9.38 | 1072.34 | 4.70 | 4261 Rosenheim | 5285367.136 | 722233.555 |
| 24 | 8.85 | 814.57 | 4.86 | 3366 Mühldorf | 5298301.368 | 780954.5959 |

S2: Climate models used for acquiring future climate data. {rcp} denotes the corresponding designation of the RCP scenarios (e.g.: {rcp26}, {rcp45}, {rcp85}).

1. ICHEC-EC-EARTH_{rcp}_r12i1p1_CLMcom-CCLM4-8-17_v1
2. ICHEC-EC-EARTH_{rcp}_r12i1p1_KNMI-RACMO22E_v1
3. ICHEC-EC-EARTH_{rcp}_r12i1p1_SMHI-RCA4_v1
4. ICHEC-EC-EARTH_{rcp}_r3i1p1_DMI-HIRHAM5_v1
5. MPI-M-MPI-ESM-LR_{rcp}1_r1i1p1_MPI-CSC-REMO2009_v1
6. MPI-M-MPI-ESM-LR_{rcp}_r2i1p1_MPI-CSC-REMO2009_v1

S3: Future (2020 – 2099) climate data acquired for the 24 scenario sites as per Fig. 4. Climate station number, name as well as site coordinates as per S1. The climate data were averaged across all 6 projections available per RCP scenario. Displayed are: Mean air temperature in 2 m (Temp.), mean annual precipitation sum (Prec.) and mean global radiation (Glob.). The data was provided by the DWD in Brunswick.

| **Site nr.** | **RCP 2.6** | | | **RCP 4.5** | | | **RCP 8.5** | | |
| --- | --- | --- | --- | --- | --- | --- | --- | --- | --- |
|  | **Temp. [°C]** | **Prec. [mm]** | **Glob. [J cm^-2^]** | **Temp. [°C]** | **Prec. [mm]** | **Glob. [J cm^-2^]** | **Temp. [°C]** | **Prec. [mm]** | **Glob. [J cm^-2^]** |
| 1 | 9.48 | 785.49 | 946.39 | 9.94 | 795.38 | 934.30 | 10.71 | 815.65 | 920.02 |
| 2 | 9.39 | 534.60 | 987.86 | 9.88 | 549.94 | 974.34 | 10.70 | 562.90 | 961.47 |
| 3 | 9.86 | 813.98 | 926.03 | 10.32 | 818.77 | 913.90 | 11.09 | 841.10 | 900.56 |
| 4 | 10.76 | 808.81 | 951.50 | 11.23 | 813.79 | 939.43 | 11.97 | 826.69 | 927.50 |
| 5 | 10.16 | 784.72 | 917.59 | 10.61 | 787.88 | 906.90 | 11.35 | 803.80 | 891.58 |
| 6 | 9.84 | 811.16 | 928.35 | 10.30 | 819.22 | 917.14 | 11.05 | 838.71 | 902.17 |
| 7 | 10.30 | 589.68 | 1030.24 | 10.80 | 602.20 | 1015.54 | 11.63 | 617.48 | 1004.66 |
| 8 | 9.96 | 552.78 | 1012.51 | 10.45 | 561.86 | 997.72 | 11.27 | 579.92 | 987.63 |
| 9 | 9.20 | 646.29 | 1009.52 | 9.69 | 659.69 | 994.43 | 10.53 | 678.74 | 985.69 |
| 10 | 8.34 | 804.40 | 993.68 | 8.81 | 820.17 | 980.01 | 9.61 | 846.81 | 969.06 |
| 11 | 8.20 | 856.08 | 999.05 | 8.71 | 878.62 | 985.96 | 9.56 | 895.30 | 976.90 |
| 12 | 9.49 | 935.61 | 995.20 | 9.97 | 953.01 | 985.14 | 10.75 | 959.00 | 976.01 |
| 13 | 8.59 | 1021.94 | 1012.47 | 9.09 | 1032.24 | 1003.15 | 9.89 | 1051.41 | 994.53 |
| 14 | 9.12 | 756.25 | 1063.43 | 9.64 | 764.46 | 1055.73 | 10.49 | 781.77 | 1047.62 |
| 15 | 8.78 | 913.51 | 1034.12 | 9.29 | 922.48 | 1023.05 | 10.12 | 944.67 | 1014.28 |
| 16 | 8.98 | 756.50 | 1119.20 | 9.51 | 767.92 | 1112.21 | 10.37 | 788.25 | 1102.81 |
| 17 | 7.92 | 1358.66 | 1065.34 | 8.46 | 1356.38 | 1060.72 | 9.33 | 1365.24 | 1055.97 |
| 18 | 7.66 | 922.67 | 1034.63 | 8.18 | 933.02 | 1025.89 | 9.03 | 955.02 | 1015.52 |
| 19 | 10.37 | 603.34 | 1006.46 | 10.86 | 610.27 | 992.99 | 11.68 | 633.70 | 981.47 |
| 20 | 9.27 | 1078.61 | 1131.97 | 9.81 | 1085.20 | 1125.41 | 10.70 | 1110.29 | 1118.75 |
| 21 | 8.94 | 877.76 | 1138.05 | 9.48 | 882.80 | 1131.18 | 10.35 | 907.77 | 1122.61 |
| 22 | 9.33 | 899.40 | 1128.89 | 9.87 | 909.87 | 1122.68 | 10.74 | 937.96 | 1112.90 |
| 23 | 6.90 | 1744.69 | 1090.67 | 7.46 | 1747.63 | 1084.50 | 8.41 | 1784.86 | 1080.10 |
| 24 | 7.47 | 1895.30 | 1090.31 | 8.03 | 1888.95 | 1083.72 | 8.98 | 1917.84 | 1077.48 |

S4a: Summary of total available climate data and share of missing data for the validation sites. Availability of climate data is reported for daily values required across the entire simulation period, thus all properties share the same amount of data entries. Missing data were acquired from the DWD Climate Data Centre [1–3].

| **Property** | Precipitation | Air temperature | Sunshine duration | **Total** |
| --- | --- | --- | --- | --- |
| Total data | 234127 | 234127 | 234127 | **702381** |
| % missing | 5.2 | 2.0 | 24.7 | **10.6** |

S4b: Summary of total available management data and share of missing data for the validation sites, with sowing date (sow. date), organic fertilizer application date (OF date) and amount (OF amt.), mineral fertilizer application date (MF date) and amount (MF amt.), cutting date (cut. date) and yield (cut. yld), grazing duration (grz. dur.) and stocking density (grz. dens.). Availability of management data is reported for the total amount of management operations documented as either dates or annual amount of operations. Due to a high correlation of the annual number of cuts, yield and fertilizer input the missing data could be completed using long-term means and descriptive management documentations of the validation sites as well as data provided by the German fertilization ordinance [4].

| **Property** | **Sow. date** | **OF date** | **OF amt.** | **MF date** | **MF amt.** | **cut. date** | **cut. yld.** | **grz. dur.** | **grz. dens.** | **Total** |
| --- | --- | --- | --- | --- | --- | --- | --- | --- | --- | --- |
| Total data | 99 | 401 | 401 | 795 | 795 | 1073 | 1073 | 769 | 769 | **6175** |
| % missing | 29.3 | 18.2 | 14.0 | 33.6 | 26.8 | 43.4 | 76.0 | 25.4 | 12.4 | **35.8** |

S4c: Summary of total available soil data and share of missing data, with rock content (rocks), soil organic carbon (SOC) content, silt and clay content, bulk density (BD), field capacity (FC), permanent wilting point (PWP), saturated hydraulic conductivity (Ks). Availability of soil data is reported per soil layer used for simulations, thus all properties share the same amount of total data entries. Missing data were acquired form the national soil map BÜK200 [5].

| **Property** | **Layer depth** | **Rocks** | **SOC** | **Silt** | **Clay** | **BD** | **FC** | **PWP** | **Ks** | **Total** |
| --- | --- | --- | --- | --- | --- | --- | --- | --- | --- | --- |
| Total data | 125 | 125 | 125 | 125 | 125 | 125 | 125 | 125 | 125 | **125** |
| % missing | 0 | 2.4 | 0 | 30.4 | 30.4 | 2.4 | 41.6 | 41.6 | 20 | **18.8** |

S5: Soil properties of the 24 scenario sites extracted from the BÜK200 soil map. Standard values were used for particle density and heat capacity. Wilting point, field capacity and saturated hydraulic conductivity were calculated with PTFs of the CANDY model as described by [6], [7] and [8]. The soil classification is provided according to the WRB [9] and the German classification guideline KA5 [10] in parentheses.

| **Site nr.** | **Lower boundary depth [dm]** | **Clay content [M-%]** | **Silt content [M-%]** | **Rock content [M-%]** | **Bulk density [g cm^-3^]** | **Soil group (topsoil)** | **Soil classification: WRB (KA5)** |
| --- | --- | --- | --- | --- | --- | --- | --- |
| 1 | 3  4  7  12  20 | 10  10  21  21  21 | 25  25  45  22.5  22.5 | 6  6  6  6  6 | 1.5  1.5  1.9  1.7  1.7 | Sand | Luvisol  (Parabraunerde) |
| 2 | 3  4  8  12  20 | 6.5  10  21  21  21 | 17.5  25  45  35  35 | 6  6  6  6  17.5 | 1.5  1.7  1.9  1.7  1.7 | Sand | Luvisol  (Parabraunerde) |
| 3 | 3  6  20 | 4  4  2.5 | 32.5  32.5  5 | 6  6  6 | 1.5  1.5  1.7 | Sand | Cambisol  (Braunerde) |
| 4 | 3  7  12  20 | 14.5  14.5  35  55 | 25  25  22.5  42.5 | 6  6  6  75 | 1.7  1.7  1.9  1.9 | Loam | Planosol  (Pseudogley) |
| 5 | 2  6  11  20 | 40  40  40  40 | 57.5  57.5  57.5  57.5 | 0  0  0  0 | 1.5  1.7  1.7  1.7 | Clay | Gleysol  (Kleimarsch) |
| 6 | 2  6  11  20 | 40  40  40  40 | 57.5  57.5  57.5  57.5 | 0  0  0  0 | 1.5  1.7  1.7  1.7 | Clay | Gleysol  (Kleimarsch) |
| 7 | 2  9  14  20 | 12.5  12.5  22.5  6.5 | 57.5  57.5  57.5  17.5 | 0  0  0  6 | 1.5  1.7  1.7  1.7 | Silt | Fluvisol  (Vega) |
| 8 | 3  6  7  10  15 | 21  21  21  12.5  35 | 74  74  74  57.5  22.5 | 0  0  0  0  17.5 | 1.5  1.5  1.7  1.7  1.7 | Silt | Chernozem  (Tschernozem) |

S5: Soil properties of the 24 scenario sites extracted from the BÜK200 soil map. Standard values were used for particle density and heat capacity. Wilting point, field capacity and saturated hydraulic conductivity were calculated with PTFs of the CANDY model as described by [6], [7] and [8]. The soil classification is provided according to the WRB [9] and the German classification guideline KA5 [10] in parentheses.

| **Site nr.** | **Lower boundary depth [dm]** | **Clay content [M-%]** | **Silt content [M-%]** | **Rock content [M-%]** | **Bulk density [g cm^-3^]** | **Soil group (topsoil)** | **Soil classification: WRB (KA5)** |
| --- | --- | --- | --- | --- | --- | --- | --- |
| 9 | 3  5  10 | 30  45  45 | 50  30  30 | 17.5  17.5  75 | 1.7  1.9  1.9 | Clay | Regosol  (Pararendzina) |
| 10 | 3  6  10 | 35  45  45 | 30  30  30 | 17.5  17.5  0 | 1.7  1.9  1.9 | Clay | Kastanozem  (Rendzina) |
| 11 | 3  6  9  12  20 | 12.5  22.5  22.5  12.5  10 | 57.5  57.5  57.5  45  25 | 62.5  62.5  62.5  75  75 | 1.7  1.7  1.9  1.9  1.9 | Silt | Cambisol  (Braunerde) |
| 12 | 1  3  5  10 | 14.5  14.5  22.5  12.5 | 76.5  76.5  57.5  57.5 | 17.5  17.5  17.5  62.5 | 1.5  1.7  1.7  1.7 | Silt | Cambisol  (Braunerde) |
| 13 | 3  5  10 | 10  10  6.5 | 25  25  17.5 | 6  17.5  37.5 | 1.5  1.7  1.7 | Sand | Cambisol  (Braunerde) |
| 14 | 3  6  10 | 6.5  6.5  30 | 17.5  17.5  40 | 0  0  0 | 1.5  1.7  1.9 | Sand | Arenosol  (Braunerde) |
| 15 | 3  4  8  12 | 30  45  65  65 | 50  30  5  5 | 17.5  17.5  17.5  75 | 1.7  1.7  1.7  1.7 | Clay | Leptosol  (Terra fusca) |
| 16 | 1  4  8  10  15 | 12.5  12.5  21  35  35 | 57.5  57.5  45  22.5  22.5 | 6  6  6  62.5  75 | 1.3  1.3  1.7  1.7  1.7 | Silt | Cambisol  (Braunerde) |

S5: Soil properties of the 24 scenario sites extracted from the BÜK200 soil map. Standard values were used for particle density and heat capacity. Wilting point, field capacity and saturated hydraulic conductivity were calculated with PTFs of the CANDY model as described by [6], [7] and [8]. The soil classification is provided according to the WRB [9] and the German classification guideline KA5 [10] in parentheses.

| **Site nr.** | **Lower boundary depth [dm]** | **Clay content [M-%]** | **Silt content [M-%]** | **Rock content [M-%]** | **Bulk density [g cm^-3^]** | **Soil group (topsoil)** | **Soil classification: WRB (KA5)** |
| --- | --- | --- | --- | --- | --- | --- | --- |
| 17 | 1  2  4  6  10 | 21  21  21  21  10 | 22.5  22.5  22.5  22.5  25 | 17.5  17.5  37.5  37.5  62.5 | 1.5  1.5  1.7  1.7  1.7 | Loam | Cambisol  (Braunerde) |
| 18 | 3  6  8  13 | 6.5  10  2.5  2.5 | 17.5  25  17.5  17.5 | 17.5  17.5  37.5  37.5 | 1.3  1.5  1.5  1.7 | Sand | Arenosol  (Braunerde) |
| 19 | 3  6  13  20 | 14.5  14.5  21  21 | 25  25  22.5  22.5 | 1  1  1  1 | 1.5  1.5  1.7  1.9 | Loam | Fluvisol  (Vega) |
| 20 | 2  4  20 | 21  40  4 | 45  40  32.5 | 17.5  37.5  75 | 1.5  1.7  1.7 | Loam | Luvisol  (Parabraunerde) |
| 21 | 3  9  10  20 | 21  21  14.5  10 | 45  35  25  25 | 6  6  6  6 | 1.7  1.7  1.7  1.7 | Loam | Cambisol  (Braunerde) |
| 22 | 2  4  8  12  20 | 30  40  40  40  30 | 40  40  57.5  57.5  70 | 0  0  0  0  0 | 1.7  1.7  1.9  2.1  1.9 | Loam | Cambisol  (Braunerde) |
| 23 | 1  20 | 4  4 | 32.5  45 | 37.5  62.5 | 1.5  1.7 | Sand | Leptosol  (Rendzina) |
| 24 | 1  3  5  20 | 40  40  55  40 | 40  40  42.5  57.5 | 6  6  1  17.5 | 1.3  1.5  1.5  1.9 | Clay | Cambisol  (Braunerde) |

S6: Summary description of management regimes developed from management data from the long-term soil survey sites and remote sensing data [11–13]. The management operations are repeated for every year within one regime. All ten regimes were applied on all scenario sites and under all climate scenarios. Mineral fertilizer input was assumed in the form of calcium ammonium nitrate (CAN), organic fertilization in form of cattle manure with a nitrogen content of 0.46 kg N ha^-1^ [14]. In accordance with the DüV [4], the annual application rate of organic fertilizers was limited to a maximum of 170 kg N ha^.1^, which corresponds do 370 dt FM ha^-1^. In order to ensure a comparability of the grazing scenarios, the grazing intensity, herein defined as the product of grazing days and number of cattle units per hectare, is kept at a constant of 50 CU ha^-1^ d.

| **Regime** | **Management practice** | **Quantity** | **Date** |
| --- | --- | --- | --- |
| 1 pasture | Start grazing End grazing | 0.2 CU ha^-1^ | 01.04. 30.11. |
| 2 meadow | Mineral fertilization Mowing | 55 kg N ha^-1^  40 dt DM ha^-1^ | 01.04. 01.08. |
| 3 mown pasture | Organic fertilization Mowing Start grazing End grazing | 220 dt FM ha^-1^ 40 dt DM ha^-1^ 0.25 CU ha^-1^ 0.25 CU ha^-1^ | 01.04. 01.05. 01.06. 30.11. |
| 4 meadow | Mineral fertilization Mowing Mineral fertilization Mowing | 65 kg N ha^-1^ 42 dt DM ha^-1^ 35 kg N ha^-1^ 13 dt DM ha^-1^ | 01.04. 01.05. 15.06. 01.09. |
| 5 mown pasture | Mineral fertilization Mowing Organic fertilization Mowing Start grazing End grazing | 125 kg N ha^-1^ 35 dt DM ha^-1^ 140 dt FM ha^-1^ 20 dt DM ha^-1^ 0.4 CU ha^-1^ 0.4 CU ha^-1^ | 01.04. 01.05. 15.05. 01.07. 01.08. 30.11. |
| 6 meadow | Mineral fertilization Mowing Mineral fertilization Mowing Mineral fertilization Mowing | 125 kg N ha^-1^ 40 dt DM ha^-1^ 32.5 kg N ha^-1^ 20 dt DM ha^-1^ 32.5 kg N ha^-1^ 20 dt DM ha^-1^ | 15.03. 01.05. 15.05. 01.07. 15.07. 01.09. |

S6: Summary description of management regimes developed from management data from the long-term soil survey sites and remote sensing data [11–13]. The management operations are repeated for every year within one regime. All ten regimes were applied on all scenario sites and under all climate scenarios. Mineral fertilizer input was assumed in the form of calcium ammonium nitrate (CAN), organic fertilization in form of cattle manure with a nitrogen content of 0.46 kg N ha^-1^ [14]. In accordance with the DüV [4], the annual application rate of organic fertilizers was limited to a maximum of 170 kg N ha^.1^, which corresponds do 370 dt FM ha^-1^. In order to ensure a comparability of the grazing scenarios, the grazing intensity, herein defined as the product of grazing days and number of cattle units per hectare, is kept at a constant of 50 CU ha^-1^ d.

| **Regime** | **Management practice** | **Quantity** | **Date** |
| --- | --- | --- | --- |
| 7 mown pasture | Mineral fertilization Mowing Mineral fertilization Mowing Organic fertilization Mowing Organic fertilization Start grazing End grazing | 165 kg N ha^-1^ 40 dt DM ha^-1^ 25 kg N ha^-1^ 20 dt DM ha^-1^ 55 dt FM ha^-1^ 20 dt DM ha^-1^ 55 dt FM ha^-1^ 0.55 CU ha^-1^  0.55 CU ha^-1^ | 01.03. 15.04. 01.05. 15.06. 01.07. 01.08. 15.08. 01.09. 30.11. |
| 8 meadow | Mineral fertilization Mowing Mineral fertilization Mowing Mineral fertilization Mowing Mineral fertilization  Mowing | 165 kg N ha^-1^ 45 dt DM ha^-1^ 40 kg N ha^-1^ 25 dt DM ha^-1^ 20 kg N ha^-1^ 10 dt DM ha^-1^ 20 kg N ha^-1^ 10 dt DM ha^-1^ | 01.03. 15.04. 01.05. 15.06. 01.07. 01.08. 15.08. 01.10. |

S6: Summary description of management regimes developed from management data from the long-term soil survey sites and remote sensing data [11–13]. The management operations are repeated for every year within one regime. All ten regimes were applied on all scenario sites and under all climate scenarios. Mineral fertilizer input was assumed in the form of calcium ammonium nitrate (CAN), organic fertilization in form of cattle manure with a nitrogen content of 0.46 kg N ha^-1^ [14]. In accordance with the DüV [4], the annual application rate of organic fertilizers was limited to a maximum of 170 kg N ha^.1^, which corresponds do 370 dt FM ha^-1^. In order to ensure a comparability of the grazing scenarios, the grazing intensity, herein defined as the product of grazing days and number of cattle units per hectare, is kept at a constant of 50 CU ha^-1^ d.

| **Regime** | **Management practice** | **Quantity** | **Date** |
| --- | --- | --- | --- |
| 9 mown pasture | Mineral fertilization Mowing Mineral fertilization Mowing Mineral fertilization Mowing Organic fertilization Mowing Organic fertilization Start grazing End grazing | 200 kg N ha^-1^ 75 dt DM ha^-1^ 27.5 kg N ha^-1^ 20 dt DM ha^-1^ 27.5 kg N ha^-1^ 10 dt DM ha^-1^ 60 dt FM ha^-1^ 5 dt DM ha^-1^ 60 dt FM ha^-1^ 0.65 CU ha^-1^ 0.65 CU ha^-1^ | 01.03. 01.04. 15.04. 15.05. 01.06. 01.07. 15.07. 15.08. 01.09. 15.09. 30.11. |
| 10 meadow | Mineral fertilization Mowing Mineral fertilization Mowing Mineral fertilization Mowing Mineral fertilization Mowing Mineral fertilization Mowing | 210 kg N ha^-1^ 70 dt DM ha^-1^ 25 kg N ha^-1^ 20 dt DM ha^-1^ 25 kg N ha^-1^ 10 dt DM ha^-1^ 25 kg N ha^-1^ 5 dt DM ha^-1^ 25 kg N ha^-1^ 5 dt DM ha^-1^ | 01.03. 01.04. 15.04. 15.05. 01.06. 01.07. 15.07. 15.08. 01.09. 15.10. |



S7: Correlation matrix of management data (n = 70) used for the simulations with the management regimes with grazing intensity (Grz_Int), stocking density (CU_ha), duration of grazing period (Grz_d), annual mineral fertilizer input (MF), annual organic fertilizer input (OF), annual yield (Yld), annual number of cuts (Cuts).





S8: Correlation matrix (n = 3888) of climate data with precipitation (P), temperature (T) and global radiation (G) aggregated by average annual means/sums (AAM), mean intra-annual variability (MIV) and inter-annual variance of means (IVM).





S9: Residual plots of GM-SP with variance structures according to soil group (top left), RCP scenario (top right), climate projection (bottom left) and management regime (bottom right).





S10: Residual plots of the best candidate models: CM-AAM-1 (top left), CM-MIV-1 (top right), CM-IVM-1 (bottom left) and CM-SP-1 (bottom right).


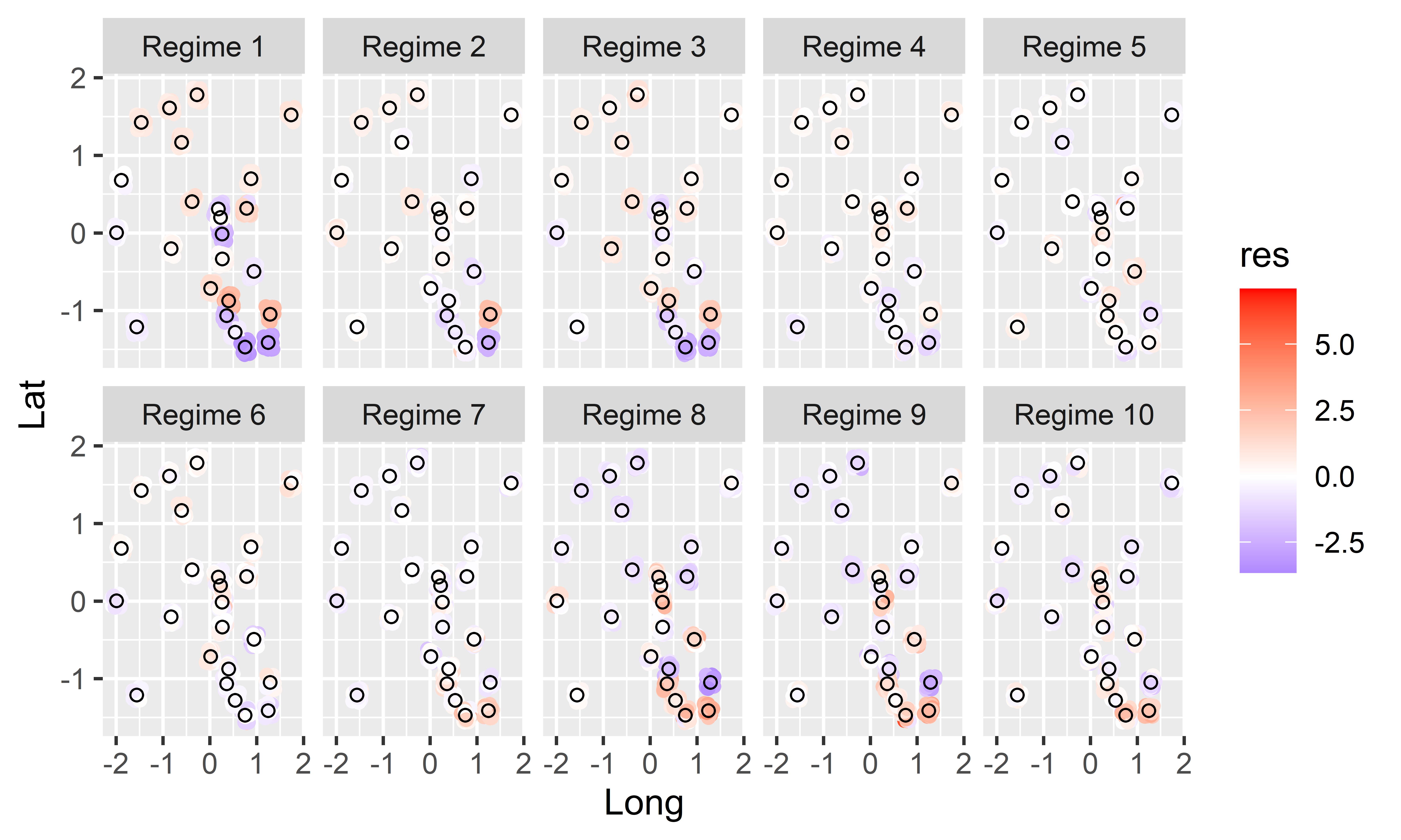
S11: Residuals of CM-SP-1 (circles with colour gradient), grouped by management regimes (1 – 10). Each black-outlined circle represents one of the 24 sites with 18 simulation results (3 RCP scenarios * 6 projections). The coordinates (longitude and latitude) of the residuals were scaled and modified by a small, random number in order to avoid visual overlap as well as issues related to null-distances when calculating Moran’s I at the site level. The graph shows a spatial clustering of sites with similar residuals, implying some spatial autocorrelation.

S12: Estimated change in SOC [M-%] per unit increase of the MIV of air temperature [°C], precipitation [mm], and global radiation [J cm^-2^] for four soil types and ten management regimes (based on CM-MIV-1 model).

| **Management regime** | **Soil group** | **SOC change [M-%] per one unit increase of MIV of** | | |
| --- | --- | --- | --- | --- |
|  |  | **Air temperature** | **Precipitation** | **Global radiation** |
| 1 | Clays  Loams  Sands  Silts | 0.02721  0.10145  -0.01342  0.09119 | -0.05756  -0.03281  -0.02823  0.02104 | 0.00024  -0.00012  0.00010  0.00093 |
| 2 | Clays  Loams  Sands  Silts | 0.09157  0.05735  -0.01594  -0.05617 | -0.04202  0.00294  0.01068  0.02320 | 0.00027  0.00009  0.00032  0.00012 |
| 3 | Clays  Loams  Sands  Silts | 0.09980  0.04483  -0.03338  0.05579 | -0.04961  -0.02245  -0.01914  0.02308 | 0.00020  -0.00016  0.00011  0.00052 |
| 4 | Clays  Loams  Sands  Silts | 0.10018  0.03934  -0.00447  -0.06032 | 0.00159  -0.01112  0.00831  0.02016 | 0.00023  0.00020  0.00019  -0.00017 |
| 5 | Clays  Loams  Sands  Silts | 0.07983  -0.00875  0.04469  0.02222 | 0.04954  0.00781  0.01128  0.04469 | 0.00019  0.00013  0.00051  0.00046 |
| 6 | Clays  Loams  Sands  Silts | 0.14227  -0.00461  -0.01249  -0.03755 | 0.01636  -0.02108  0.00534  0.01005 | -0.00019  0.00020  0.00049  0.00030 |
| 7 | Clays  Loams  Sands  Silts | 0.06116  0.01673  0.02216  -0.02249 | 0.09877  -0.00324  0.02951  0.01318 | 0.00024  0.00059  0.00060  0.00046 |
| 8 | Clays  Loams  Sands  Silts | 0.16949  -0.04017  0.05814  -0.15721 | 0.13374  0.01882  0.02508  0.01318 | -0.00014  0.00074  0.00076  -0.00008 |
| 9 | Clays  Loams  Sands  Silts | 0.09879  -0.02125  0.07005  -0.08815 | 0.13454  0.01108  0.02786  0.02637 | 0.00019  0.00064  0.00068  0.00012 |
| 10 | Clays  Loams  Sands  Silts | 0.17561  -0.03205  -0.02412  -0.10161 | 0.10450  0.00899  0.02360  -0.02166 | -0.00025  0.00067  0.00073  0.00040 |

S13: Simulated change in SOC [M-%] acquired from the CANDY model in dependence of climate projection (model designations according to supplementary material S2) and RCP scenario, with management regimes as greyscale. Climate projections change row-wise, RCP scenarios column-wise. The 24 scenario sites are spread along the x-axis, numbered from left to right in accordance to Fig. 4. The graph shows a) visible differences in the SOC balance between different sites, b) small differences between different climate projections and c) large differences between management regimes, thereby substantiating a low uncertainty due to climate data.





References

[1] DWD Climate Data Centre, Daily station observations of mean temperature at 2 m above ground in °C for Germany, version 19.3, 2021. <https://cdc.dwd.de/portal/202102121428/mapview>.

[2] DWD Climate Data Centre, Daily station observations of precipitation height in mm for Germany, version 19.3, 2021. <https://cdc.dwd.de/portal/202102121428/mapview>.

[3] DWD Climate Data Centre, Daily station observations of sunshine duration in hours for Germany, version 19.3, 2021. <https://cdc.dwd.de/portal/202102121428/mapview>.

[4] DüV, Verordnung über die Anwendung von Düngemitteln, Bodenhilfsstoffen, Kultursubstraten und Pflanzenhilfsmitteln nach den Grundsätzen der guten fachlichen Praxis beim Düngen (Düngeverordnung - DüV), 2017.

[5] BGR Boden, BÜK200: Bodenübersichtskarte 1:200.000, Bundesanstalt für Geowissenschaften und Rohstoffe, Hannover, 2018.

[6] U. Franko, B. Oelschlägel, S. Schenk, Simulation of temperature-, water- and nitrogen dynamics using the model CANDY, Ecological Modelling 81 (1995) 213–222. <https://doi.org/10.1016/0304-3800(94)00172-E>.

[7] U. Franko, Modelling approaches of soil organic matter turnover within the CANDY system, in: Evaluation of Soil Organic Matter Models, Berlin, Heidelberg, Springer Berlin Heidelberg, Berlin, Heidelberg, 1996, pp. 247–254.

[8] U. Franko, G.J. Crocker, P.R. Grace, J. Klír, M. Körschens, P.R. Poulton, D.D. Richter, Simulating trends in soil organic carbon in long-term experiments using the CANDY model, Geoderma 81 (1997) 109–120. <https://doi.org/10.1016/S0016-7061(97)00084-0>.

[9] IUSS Working Group WRB, World Reference Base for Soil Resources: International soil classification system for naming soils and creating legends for soil maps, fourthth, Vienna, Austria, 2022.

[10] H. Sponagel, Bodenkundliche Kartieranleitung: [KA 5] ; mit 103 Tabellen und 31 Listen, fifth., verb. u. erw. Aufl., Schweizerbart, Stuttgart, 2005.

[11] P. Griffiths, C. Nendel, J. Pickert, P. Hostert, Towards national-scale characterization of grassland use intensity from integrated Sentinel-2 and Landsat time series, Remote Sensing of Environment 238 (2020) 111124. <https://doi.org/10.1016/j.rse.2019.03.017>.

[12] P. Griffiths, C. Nendel, P. Hostert, Intra-annual reflectance composites from Sentinel-2 and Landsat for national-scale crop and land cover mapping, Remote Sensing of Environment 220 (2019) 135–151. <https://doi.org/10.1016/j.rse.2018.10.031>.

[13] P. Griffiths, C. Nendel, P. Hostert, National-scale crop- and land-cover map of Germany (2016) based on imagery acquired by Sentinel-2A MSI and Landsat-8 OLI, PANGAEA, 2018.

[14] LWK Niedersachsen, Nährstoffgehalte in organischen Düngern: Richtwerte für organische Wirtschaftsdünger (2018).
